# The encapsulation selectivity for anionic fission products imparted by an electride

**DOI:** 10.1038/s41598-019-50089-0

**Published:** 2019-09-20

**Authors:** Navaratnarajah Kuganathan, Alexander Chroneos, Robin W. Grimes

**Affiliations:** 1 Department of Materials, Imperial College London, London, SW7 2AZ UK; 2 Faculty of Engineering, Environment and Computing, Coventry University, Priory Street, Coventry, CV1 5FB UK

**Keywords:** Density functional theory, Carbon capture and storage

## Abstract

The nanoporous oxide 12CaO·7Al_2_O_3_ (C12A7) can capture large concentrations of extra-framework species inside its nanopores, while maintaining its thermodynamical stability. Here we use atomistic simulation to predict the efficacy of C12A7 to encapsulate volatile fission products, in its stoichiometric and much more effective electride forms. In the stoichiometric form, while Xe, Kr and Cs are not captured, Br, I and Te exhibit strong encapsulation energies while Rb is only weakly encapsulated from atoms. The high electronegativities of Br, I and Te stabilize their encapsulation as anions. The electride form of C12A7 shows a significant enhancement in the encapsulation of Br, I and Te with all three stable as anions from their atom and dimer reference states. Successive encapsulation of multiple Br, I and Te as single anions in adjacent cages is also energetically favourable. Conversely, Xe, Kr, Rb and Cs are unbound. Encapsulation of homonuclear dimers (Br_2_, I_2_ and Te_2_) and heteronuclear dimers (CsBr and CsI) in a single cage is also unfavourable. Thus, C12A7 offers the desirable prospect of species selectivity.

## Introduction

Volatile radioactive species generated during the processing of spent nuclear fuel must be disposed of carefully to prevent contamination of the biosphere. The volatile species include chemically inert noble gases (Kr and Xe), alkali metals (Rb and Cs) and halogens (Br and I)^1^. Among these, radioactive iodine is particularly challenging as it is highly mobile^2^, has high abundance in the spent fuel^3^ and can be concentrated by the body, increasing the radiotoxic impact. Caesium is also a particularly hazardous radio nucleotide but has a much longer half-life^4^. Therefore, separating iodine and caesium would be beneficial.

Impregnated carbon filters including charcoal filters are mostly used to remove iodine species in nuclear power plants^5^. Considerable effort has been devoted to modify and improve the effectiveness of filters using new materials. Porous materials including zeolites, silica, alumina and metal organic frameworks have been studied as these materials provide higher capacity to incorporate iodine^5–9^. The search for alternative filter materials exhibiting selectivity, thermodynamical stability, chemical activity, adequate mechanical properties and high capacity to incorporate guest nucleotides continues, while it is still a requirement they be manufactured at scale in a cost-effective manner.

Mayenite, a nanoporous complex inorganic oxide 12CaO·7Al_2_O_3_ (C12A7)^10–12^, and a constituent of aluminous cement^13^, is a candidate material for trapping volatile fission products because of its open crystal structure (consisting of 12 subnanometer-sized cages in a unit cell), but also its chemical and thermal stability^12^. The diameter of the cage inner free space is ~0.4 nm^12^. Neighboring cages are interconnected via ~0.1 nm wide openings. Furthermore, its constituent metal oxides (Al_2_O_3_ and CaO) are highly abundant, low cost and non-toxic. The framework of C12A7 is represented using the chemical formula [Ca_24_Al_28_O_64_]^4+^, in which each cage bears a charge of +1/3. The positive charge of the framework is compensated by negative ions occupying some of the cages; two O^2−^ ions in stoichiometric C12A7 (C12A7:O^2−^)^10,11^, four OH^−^ ions in fully hydrated C12A7: OH^−^ ^14,15^, and four electrons in the electride form, C12A7 (C12A7:e^−^)^16,17^.

Due to its extra-framework electrons, C12A7:e^−^ has the ability to accept and release electrons when guest species are present inside its cages. The extra-framework electrons can be replaced with other anions such as F^−^ ^18^, Cl^−^ ^18^, H^−^ ^15^, S^2–19^, O_2_^−^ ^20^ and OH^−^ ^14,15^. Transition metals such as Au^21^ and Ru^22,23^ have also been incorporated in order to form catalysts (for example Ru in the synthesis of ammonia).

Using density functional theory (DFT) together with dispersion corrections (DFT + D), the thermodynamic stability of gaseous volatile fission products (Kr, Xe, Br, I, Rb, Cs and Te) is investigated in both stoichiometric and electride forms of C12A7. DFT calculations, in addition to generating structural information, provides electronic structure and properties information. The present calculations consider fission products accommodated in the form of single atoms, homonuclear and heteronuclear dimers.

## Results and Discussion

### Structures and electronic properties of C12A7:O^2−^ and C12A7:e^−^

**Figure 1:**
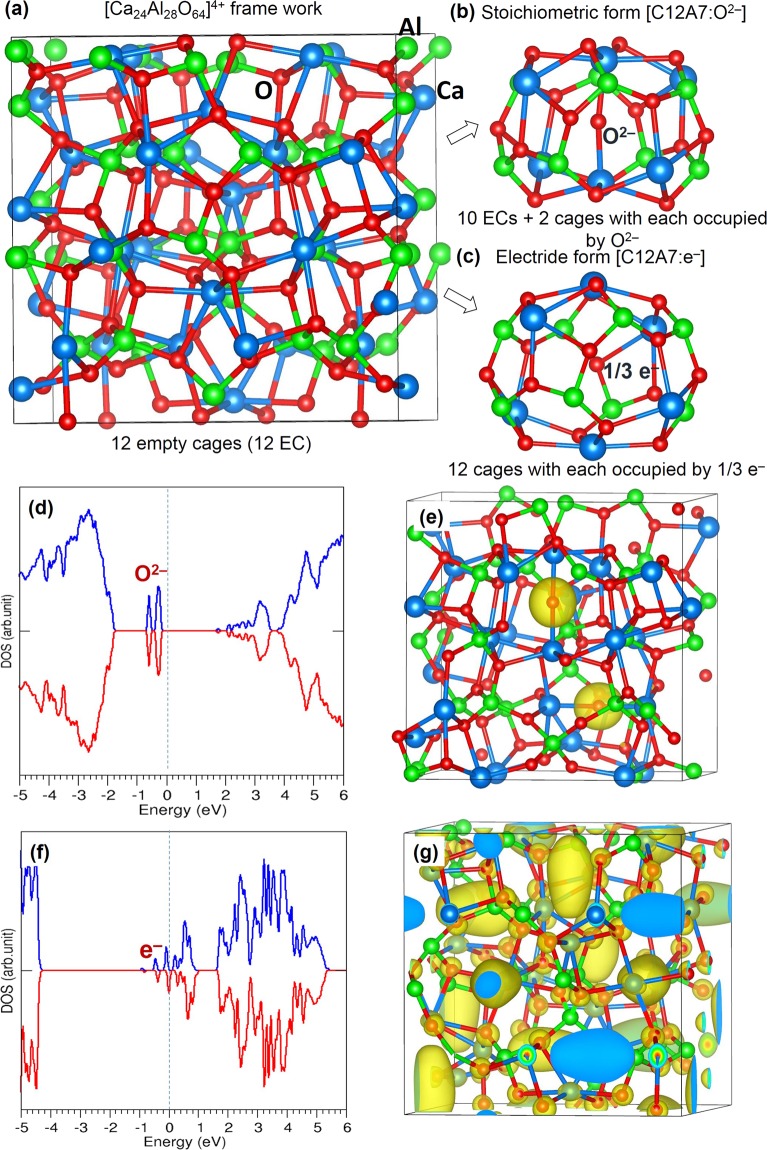
(**a**) Framework crystal structure of [Ca_24_Al_28_O_64_]^4+^ (**b**) a relaxed cage containing an O^2−^ ion in C12A7:O^2−^ (**c**) a relaxed cage containing 1/3 electron in C12A7:e^−^ (**d**) DOS plot calculated for C12A7: O^2−^ (**e**) surface of the constant charge density owing to two extra-framework O^2−^ ions (**f**) DOS plot calculated for C12A7:e^−^ and (**g**) surface of the constant charge density owing to four electrons. Vertical dotted lines in the DOS plots correspond to the Fermi energy.

C12A7 exhibits a cubic crystal structure with space group of ${\rm{I}}\bar{4}$3d and a lattice constant of 11.99 Å^11^. The unit cell contains two 12CaO·7Al_2_O_3_ molecules (118 atoms) and twelve cages. The cages are approximately 6 Å wide and each cage wall is formed by 16 O ions, 8 Al ions and 6 Ca ions. The chemical composition of the unit cell is represented by [Ca_24_Al_28_O_64_]^4+^·(O^2−^)_2_, where [Ca_24_Al_28_O_64_]^4+^ is a positively charged framework (see Fig. 1a) consisting of 12 subnanometer cages and two extra-framework oxide ions occupying two of these cages (see Fig. 1b). The electride form of C12A7 is represented by [Ca_24_Al_28_O_64_]^4+^·(e^−^)_4_ and the four electrons are localized in cages with the average occupation number of $\frac{1}{3}$ electrons per cage (see Fig. 1c).

The starting point of the study was to reproduce the experimentally observed crystal structure of C12A7:O^2−^. The structure was first relaxed under constant pressure conditions allowing both lattice parameters and ion positions to relax. The calculated equilibrium lattice parameters (a = 12.04 Å, b = c = 12.01 Å, α = 90.02°, β = 89.95° and γ = 89.93°) are in good agreement with experimental lattice parameters (a = b = c = 11.99 Å and α = β = γ = 90°)^11^ within a margin of ~0.45% error.

The relaxed structure of the cage containing the O^2−^ ion (Fig. 1b) is slightly different to cages without O^2−^ ions. This is a result of the interaction between the extra-framework O^2−^ ion and the two Ca^2+^ ions at the cage poles (see Fig. 1b). The small departure from the experimental cubic structure is due to an imposed long-range ordering of O^2−^ ions due to occupation by O^2−^ in specific cells of the unit cell. The distortion from cubic symmetry is so small this clearly an acceptable approximation. The calculated density of states is consistent with C12A7:O^2−^ being an insulator (see Fig. 1d). The top of the valence band, formed by the 2*p* states of framework oxide ions, is at approximately −2 eV. The two peaks at −0.3 eV correspond to the 2*p* states of extra-framework oxide ions. The lower energy and higher energy peaks correspond to the 2*p* states interacting with two Ca^2+^ ions (along the bonding) and the 2*p* states perpendicular to the bonding (non-bonded) respectively. Figure 1e shows the spatial distribution of the charge density associated with two extra-framework oxide ions localized within the cages.

Next, the structure of C12A7:e^−^ was relaxed under constant pressure. The calculated equilibrium lattice parameters were a = b = c = 12.06 Å and α = β = γ = 90°. Thus, the lattice volume of C12A7:e^−^ is only slightly greater than that of C12A7:O^2−^. The calculated density of states (DOS) for C12A7:e^−^ is shown in Fig. 1f. The extra-framework electrons in C12A7:e^−^ means the system is metallic. The spatial distribution of the charge density associated with the extra-framework electrons localized in the cages is shown Fig. 1g. Twelve ellipsoid like iso-surfaces (each having 1/3 e^−^ to make four electrons in total) are distributed uniformly within the 12 cages. The electronic properties calculated for both C12A7:O^2−^ and C12A7:e^−^ are in good agreement with the previous theoretical study^24^.

### Encapsulation of single fission product atoms in the cages of C12A7:O^2−^

**Figure 2:**
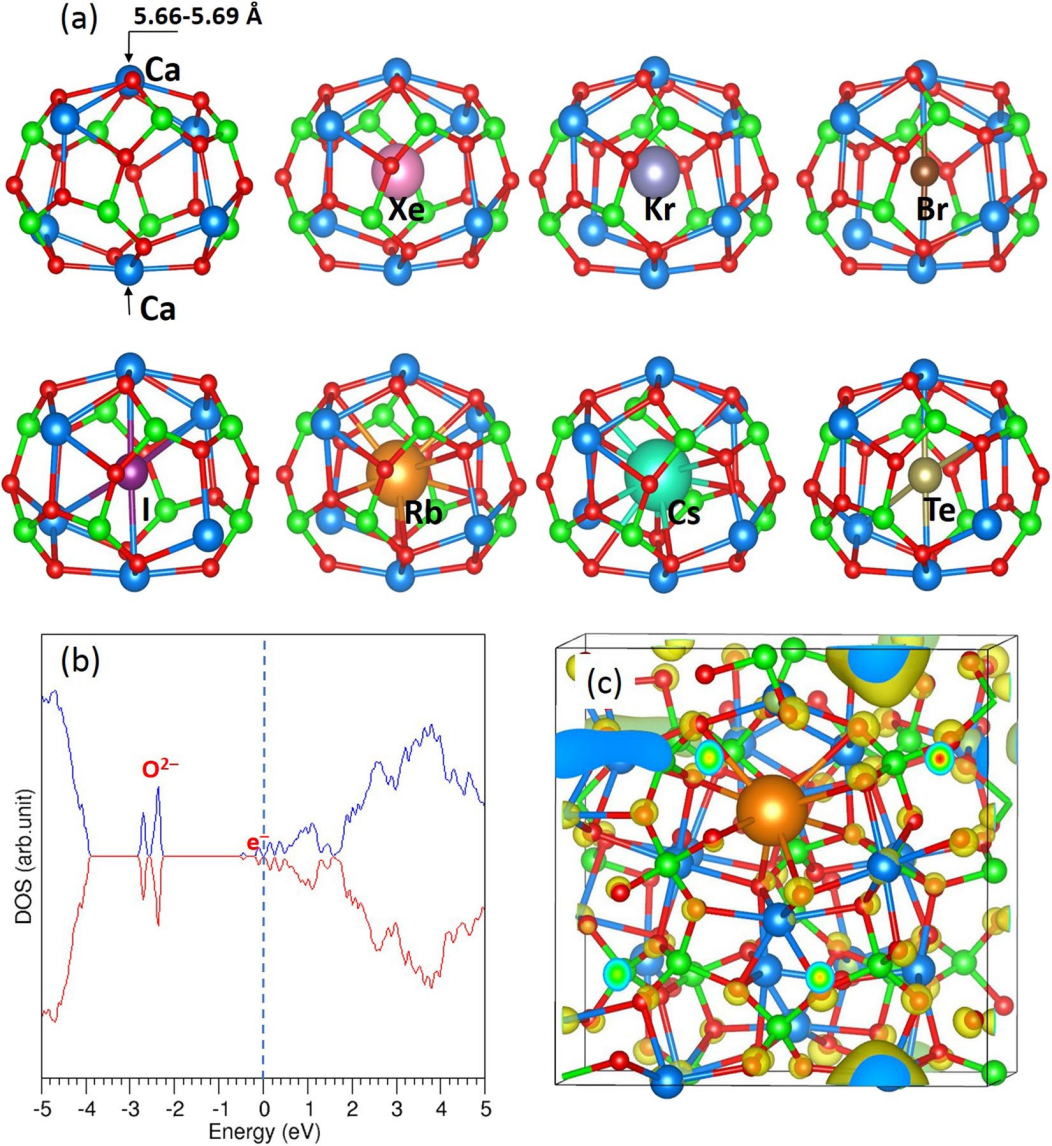
(**a**) Relaxed structures of fission atoms occupying a cage of C12A7:O^2−^, (**b**) DOS plot calculated for Rb encapsulated in an empty cage of C12A7:O^2−^ and (**c**) surface of the constant charge density owing to the s^1^ electron transferred from Rb.

To establish the stability of volatile fission products inside C12A7:O^2−^, a single atom was incorporated into one of the ten empty cages in a supercell and encapsulation energies were calculated using a single atom reference state. Figure 2 shows the relaxed structures of the cage containing a fission product atom. In all cases, atoms occupy the center of the cage.

Both Xe and Kr demonstrate positive encapsulation energies with unmodified outer electronic configurations (refer to the Bader charge in Table 1) meaning that they are unstable inside the cages. This is further supported by the non-bonding interaction with the cage wall (see Fig. 2a). The more unfavorable encapsulation energy for Xe is due to its larger size compared to Kr and this is reflected in the greater Ca-Ca cross-cage distance as defined in Fig. 2a and reported in Table 1.

**Table 1:** Encapsulation energies in C12A7:O^2−^ calculated using the fission product atom or dimer as the reference state, Bader charges on encapsulated atoms and Ca-Ca distances (see Fig. 2) across the relaxed cages occupied by the atoms.

Properties	Fission Product						
	Xe	Kr	Br	I	Te	Rb	Cs
Encapsulation energy (eV/atom) with respect to a single atom reference	0.85	0.31	−2.73	−1.56	−0.62	−0.13	+0.21
Encapsulation energy (eV/atom) with respect to dimer for Br, I and Te	—	—	−1.47	−0.44	+1.14	—	—
Bader charge |e|	+0.01	−0.02	−0.75	−0.68	−0.49	+0.80	+0.79
Ca-Ca distance (Å)	6.00	5.90	5.66	5.87	5.91	6.11	6.16
Electron affinity (eV) (atoms)^32^	0.00	0.00	3.364	3.059	1.971	0.486	0.472
Electron affinity (eV)(dimer)^32^	—	—	2.550	2.550	1.920	0.498	0.469

The encapsulation energies for Br and I are negative (favourable) due to their strong electrostatic interaction with Ca^2+^ ions. Br and I atoms assume charges of 0.75 and 0.68 |e| respectively (see Table 1). The encapsulation energy for Br is more favorable than that of I due to the higher electron affinity of Br as well as its smaller atomic radius (thus fitting better inside the cage). These are reflected in the slightly smaller Bader charge on the I and the longer Ca-Ca distance (see Table 1).

The encapsulation energy calculated using the dimer as the reference indicates that even Br_2_ molecules favourably occupy (separate) cage sites as Br^−^ ions (i.e. with an energy gain) despite the molecular dissociation energy penalty. Conversely both I and Te are not stable inside cages from their dimers. The calculated dissociation energies (per atom) for Br_2_, I_2_ and Te_2_ in this study are 1.26 eV, 1.12 eV and 1.76 eV respectively. The stronger encapsulation energy of −1.47 eV for Br is thus due to the favourable balance of exothermic encapsulation energy (−2.73 eV) over the endothermic energy penalty for Br_2_ bond dissociation. In the case of I, a less negative but still favourable encapsulation energy (−0.44 eV) is observed. This is due to the slightly less exothermic encapsulation energy (−1.56 eV) for I, balance over its dissociation energy.

The negative encapsulation energy for tellurium reveals that it is more stable inside the cage than as an isolated atom. This is due to the electron gain (0.49 |e|) by the Te and then the electrostatic attraction to Ca^2+^ ions in the cage wall (see Fig. 2). However, Te exhibits a highly unfavorable encapsulation energy from its dimer due to the high Te_2_ dissociation energy (+1.76 eV) compared to its exothermic encapsulation energy of (−0.62 eV).

Rubidium exhibits a slightly negative (favourable) encapsulation energy, whereas for Cs the energy is slightly unfavorable. Both Rb and Cs transfer their single outer s-electrons to the lattice and exhibit a positive charge (see Table 1). The slightly negative encapsulation energy for Rb is due to its smaller size compared to Cs which is reflected by the shorter Rb-O v’s Cs-O distance, 2.98 Å v’s 3.40 Å respectively. The DOS plot in Fig. 2b shows the s electron transferred from Rb occupies a state just below the Fermi energy and is localized in an empty cage as shown in Fig. 2c.

Next we considered up to 3 atoms (Br, I or Te) occupying separate cages (i.e. two of the ten initially empty cages contain a single gas atom and then three of the ten cages contain a gas atom). Calculations reveal that the encapsulation energy of a second atom (X:XC12A7:O^2−^) with respect to a single isolated species is negative but slightly smaller in magnitude compared to the first incorporation. Further, the encapsulation of a second Br is still strongly negative with respect to its dimer while the I:IC12A7:O^2−^ energy is close to zero and the Te:TeC12A7:O^2−^ energy is positive. The Bader analysis shows that the second Br or I gains the same charge as observed in the first encapsulation. Conversely, the second Te gains a smaller amount of charge.

For all three species encapsulation of the third anion, while still favourable with respect to atoms, is less favourable. Thus, encapsulation of a third I with respect to I_2_ is now not favourable though a third Br from Br_2_ remains favourable. The amount of charge gained by these third anions is also less though only slightly.

### Encapsulation of single fission products in C12A7:e^−^

**Figure 3:**
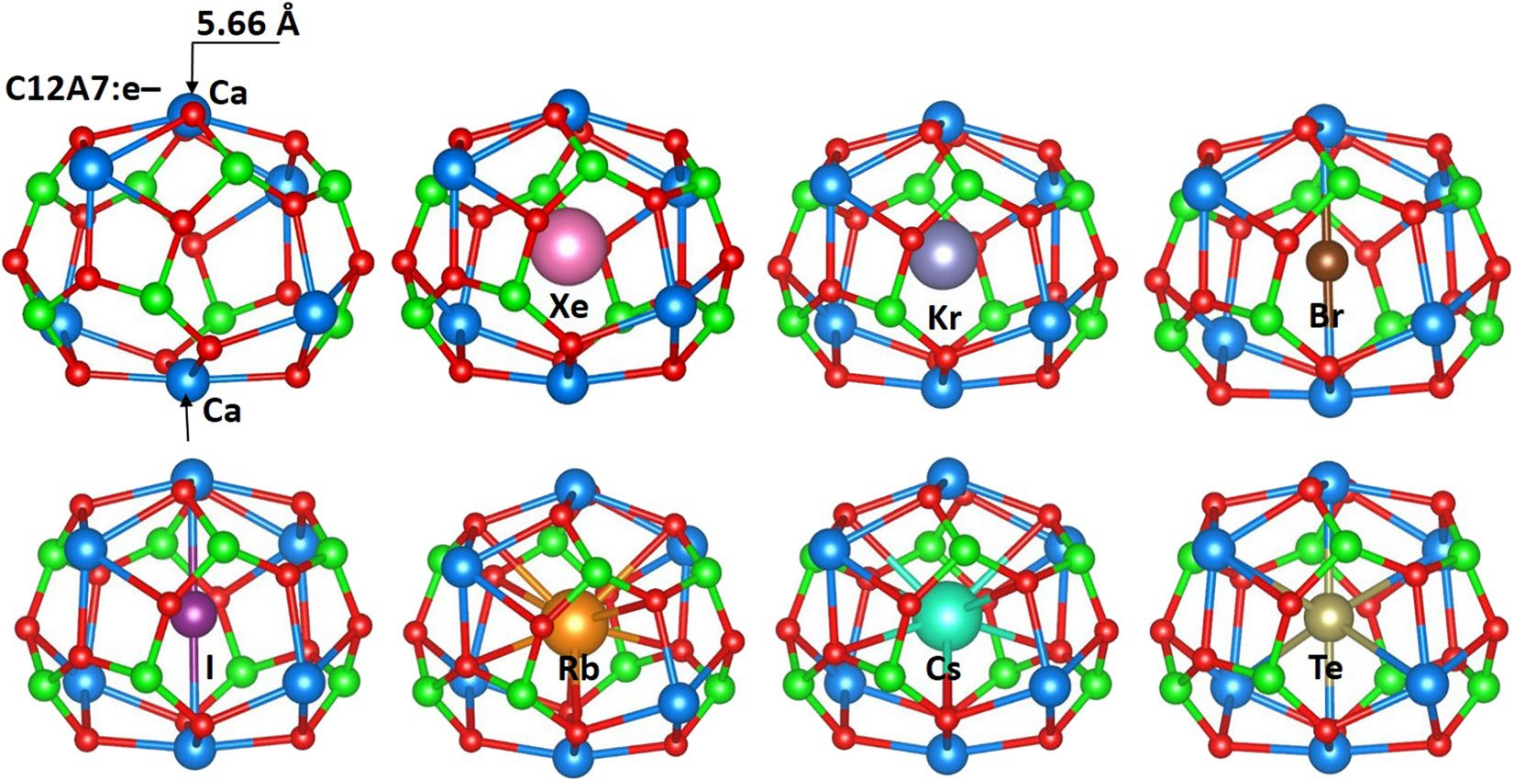
Cage structure and local atomic structures of fission products encapsulated in a C12A7:e^−^ cage.

The relaxed configurations of fission products (Xe, Kr Br, I, Cs, Rb and Te) as single atoms occupying a cage in C12A7:e^−^ are shown in the Fig. 3. All the atoms occupy positions close to the center of the cage (between two Ca ions in the cage wall). In order to compare the deformation of the occupied cage with the unoccupied cage, Ca-Ca distances (as defined in Fig. 3) for all relaxed structures are reported in Tables 2 and 3; the distance in the unoccupied cage is 5.66 Å.

**Table 2:** Encapsulation energies in C12A7:O^2−^ to add a 2^nd^ and then 3^rd^ fission atom into separate cages using both atoms and dimers as reference states. Bader charges on atoms are also reported.

Properties	Fission product X	X:XC12A7:O^2−^	X:2XC12A7:O^2−^
Encapsulation energy (eV/atom) with respect to atom	Br	−2.30	−1.62
	I	−1.16	−0.65
	Te	−0.55	−0.53
Encapsulation energy (eV/atom) with respect to dimer (½ X_2_)	Br	−1.04	−0.32
	I	−0.04	0.47
	Te	1.21	1.23
Bader charge |e|	Br	−0.75 (2)	−0.73 (2), −0.72
	I	−0.68 (2)	−0.55, −0.60 (2)
	Te	−0.45, −0.19	−0.40, −0.28, −0.20

**Table 3:** Encapsulation energies in C12A7:e^−^ calculated using the fission product atom and the dimer molecule as reference states, Bader charge on atoms and Ca-Ca distances in the relaxed cages occupied by the atoms.

Properties	Volatile Fission Products						
	Xe	Kr	Br	I	Te	Rb	Cs
Encapsulation energy (eV/atom) with respect to atom	1.00	0.43	−5.17	−3.99	−5.35	0.29	0.67
Encapsulation energy (eV/atom) with respect to dimer for Br, I and Te	—	—	−3.91	−2.87	−3.59	—	—
Bader charge |e|	+0.01	+0.04	−0.76	−0.69	−1.23	+0.78	+0.77
Ca-Ca distance (Å)	6.04	5.95	5.71	5.91	5.83	6.15	6.20

Xe exhibits a high positive encapsulation energy because of its large size and is non-bonding with the cage. This is supported by the almost zero Bader charge (refer to Table 3). The elongation of the Ca-Ca distance in the cage occupied by the Xe is 0.38 Å (compared to the empty cage) reflecting the size of Xe. Kr, in comparison, while still exhibiting a positive encapsulation energy is considerably less unfavourable due to its smaller size and thus reduced cage elongation distance of 0.29 Å. Comparing encapsulation in C12A7:e^−^ with C12A7:O^2−^, the energies and distances are essentially the same.

The encapsulation energies of Br and I in C12A7:e^−^ are highly exothermic with respect to isolated atoms. Both atoms gain one electron from the extra-framework electrons localized in the cages and form Br^−^ and I^−^ respectively. The negative ions then form strong electrostatic interactions with the two Ca^2+^ ions of the cage wall as they did in C12A7:O^2−^. Again the encapsulation energy of Br is more favorable than that of I. However, the encapsulation energies in C12A7:e^−^ are ~2.50 eV more favourable than in C12A7:O^2−^. Consequently, the encapsulation energy calculated using ½ X_2_ (X = Br, I and Te) as reference is strongly exothermic. This is because of the electron affinities of Br and I (3.36 eV and 3.06 eV respectively)^25^ which are gained when one of the extra-framework electrons complete the p-shells of these atoms.

Te is also predicted to exhibit a highly negative encapsulation energy. Indeed, its Bader charge is -1.23 electrons meaning that it is tending towards a Te^2−^ ion, close to completing its p-shell. Thus, unlike in C12A7:O^2−^, in C12A7:e^−^ Te is stable in a cage with respect to dimer dissociation.

Both Rb and Cs exhibit slightly positive encapsulation energies (0.29 eV and 0.67 eV respectively). Bader charge analysis shows that both Rb and Cs donate ~one electron to form the +1 charge state. Once the positive ions are formed, they are attracted by the cage wall O^2−^ ions (refer to Fig. 3), however, they are repelled by the Ca^2+^ ions as reflected in the longer Ca-Ca distance (see Table 3). Compared to encapsulation in C12A7:O^2−^ these energies are ~0.45 eV higher.

**Figure 4:**
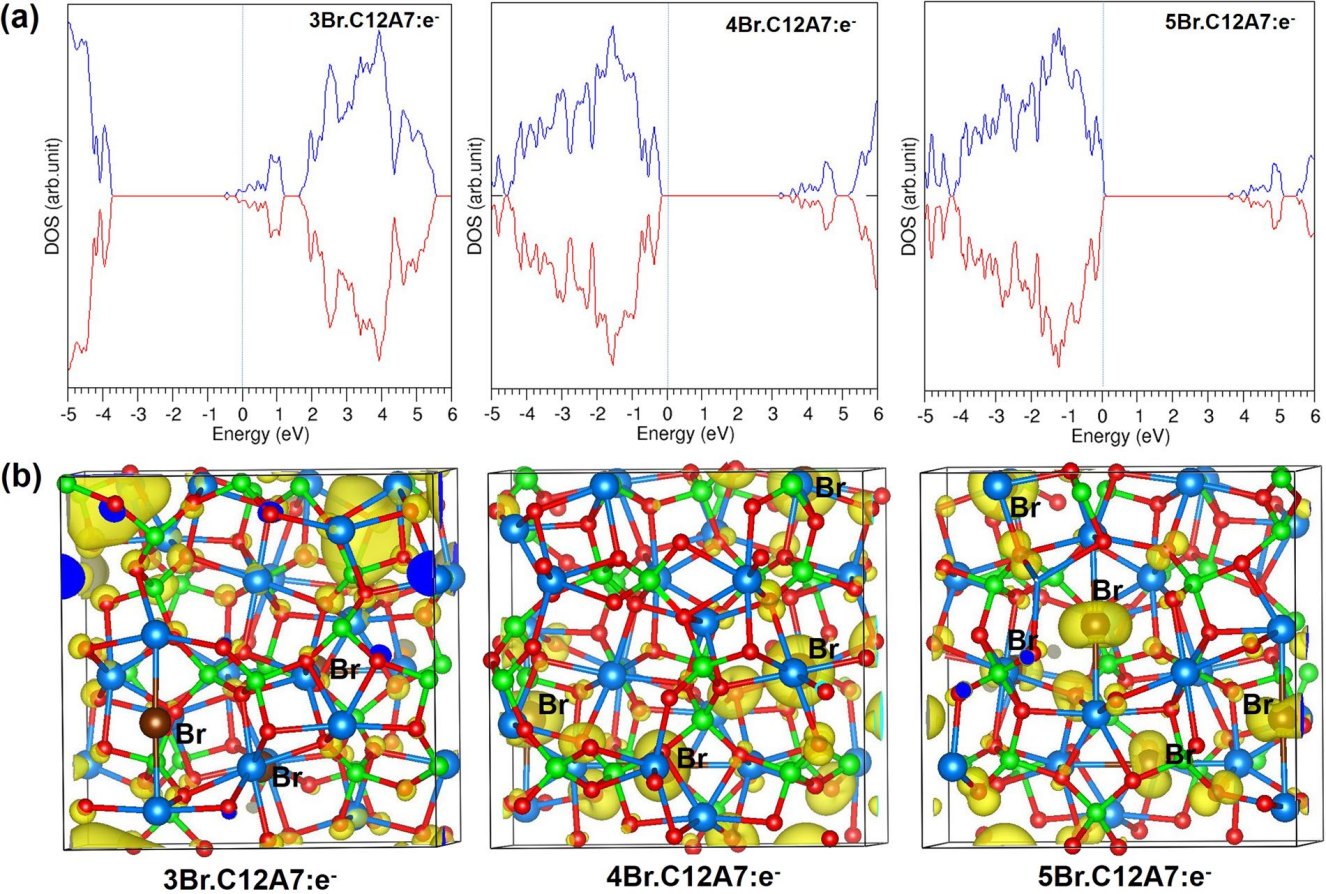
(**a**) DOS plots for 3Br, 4Br and 5Br encapsulated in C12A7:e^−^ with Fermi level indicated by vertical lines and (**b**) surface of the constant charge density associated with the sates below the Fermi energy.

Successive encapsulation was considered for up to 5 atoms of Br, I and Te inside separate unoccupied cages. Both Br and I show almost no change in the favourable encapsulation energies up to 4 atoms (see Table 4). This is because there are four conduction electrons available in the repeat unit lattice. The Bader charge analysis show that both Br and I gain similar charges up to four successive atoms. While the fifth halide atom also attracts only slightly less charge, there is a significant reduction in the encapsulation energy. This is because there are no free electrons left after encapsulation of four halide atoms and the charge is taken from the framework. As a consequence there is a significant Fermi level shift to the top of the valence band for the encapsulation of four Br atoms and the system becomes insulating (see Fig. 4a). This is further illustrated in Fig. 4b, which shows charge density localization within the unit cell change across the 3BrC12A7:e^−^, 4BrC12A7:e^−^, 5BrC12A7:e^−^ series. In 3BrC12A7:e^−^ considerable charge resides in empty cages but this has localised onto Br^−^ ions in 4BrC12A7:e^−^. The multiple anion encapsulation energies contrast strongly with the equivalent results in C12A7:O^2−^ where even the encapsulation of a second halide came with a sharp fall in energy.

**Table 4:** Encapsulation energies and Bader charges for the successive encapsulations of Br, I and Te in C12A7: e^−^.

Properties	Fission product X	X:XC12A7:e^−^	X:2XC12A7:e^−^	X:3XC12A7:e^−^	X:4XC12A7:e^−^
Encapsulation energy (eV/atom) with respect to atom	Br	−5.18	−5.18	−5.04	−1.39
	I	−4.06	−4.03	−3.94	−0.69
	Te	−5.32	−1.13	−0.87	−0.76
Encapsulation energy (eV/atom) with respect to dimer (½ X_2_)	Br	−3.92	−3.92	−3.78	−0.13
	I	−2.94	−2.91	−2.82	0.43
	Te	−3.56	0.63	0.89	1.00
Bader charge |e|	Br	−0.76 (2)	−0.76 (3)	−0.76 (4)	−0.69 (3), −0.67 (2)
	I	−0.69 (2)	−0.69 (3)	−0.69 (4)	−0.57, −0.60, −0.62 (3)
	Te	−1.23 (2)	−0.85, −0.87 (2)	−0.71, −0.68 (3)	−0.59 (3), −0.57 (2)

**Figure 5:**
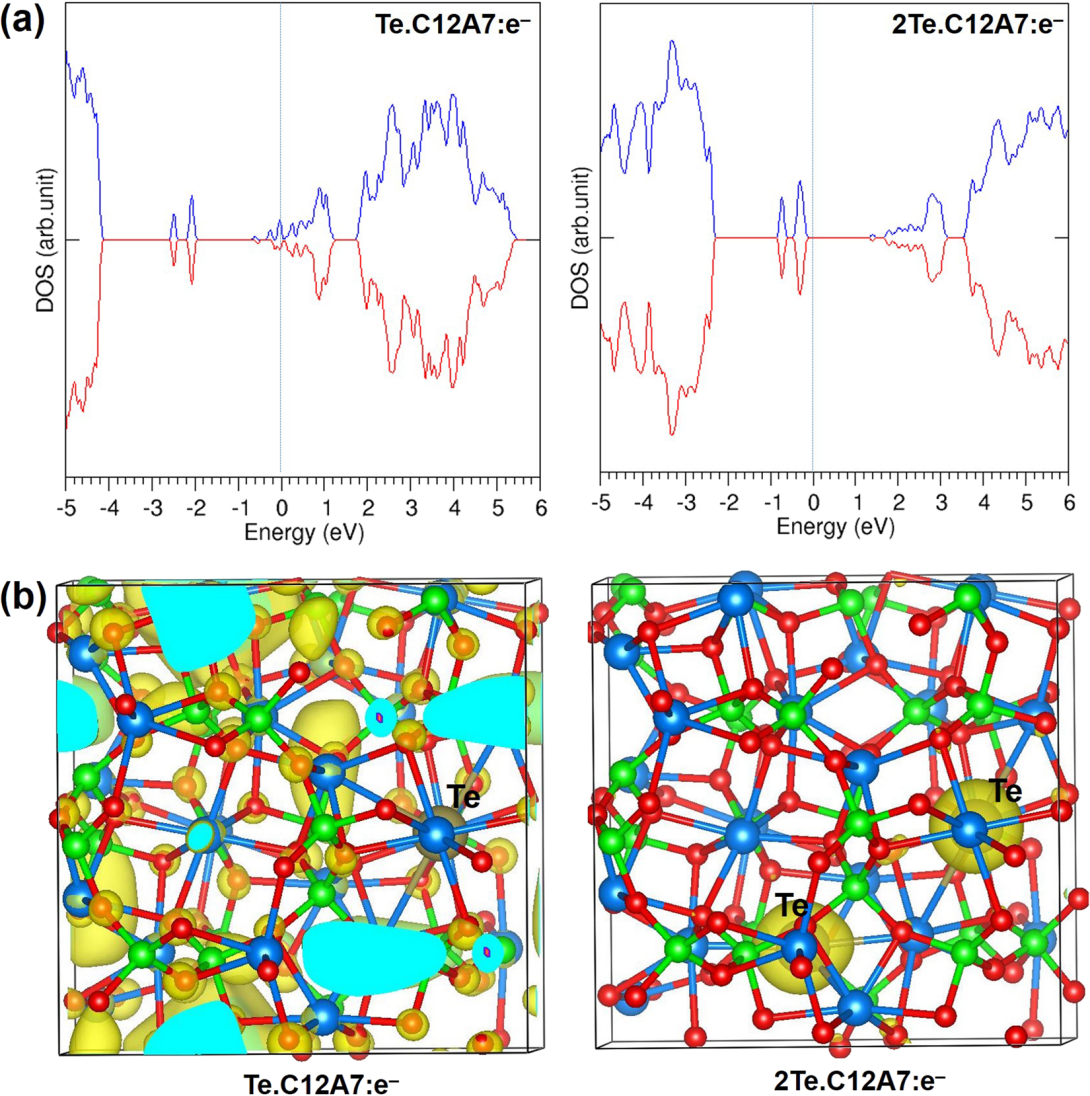
(**a**) DOS plots for Te and 2Te encapsulated in C12A7:e^−^ and (**b**) surface of the constant charge density associated with the states below the Fermi energy.

For Te, a significant reduction in the encapsulation energy is observed for the third Te atom. This is because the first two Te atoms gain almost as much charge as four Br or I atoms. Thus the third Te atom has no extra-framework electrons to exploit. When the number of Te atoms is greater than two the Bader charges on each Te atom decrease with the equivalent charge gained by two Te atoms shared across more Te atoms. Consequently, the Fermi level shifts to lower energies for the second encapsulation and the system becomes an insulator (see Fig. 5a). The DOS plotted for a single Te reveals that the system is metallic as two extra-framework electrons remain while the other two electrons are localized on the Te forming a Te^2−^ ion; the corresponding peak for the Te^2−^ ion appears −2.50 eV below the Fermi level (see Fig. 5a). When there are two Te atoms the peaks corresponding to the localized Te states are just below the Fermi level.

### Encapsulation of homonuclear and heteronuclear dimers

Calculations were performed to investigate the energetics associated with placing a single homonuclear or heteronuclear diatomic molecule within a single cage of C12A7:O^2−^ and C12A7:e^−^ (i.e. encapsulation of the molecule using the molecule as the reference states but normalized per atom). Only the reactive fission products (Br_2_, I_2_ and Te_2_) were considered as homonuclear species.

### Dimers occupying C12A7:O^2−^

**Figure 6:**
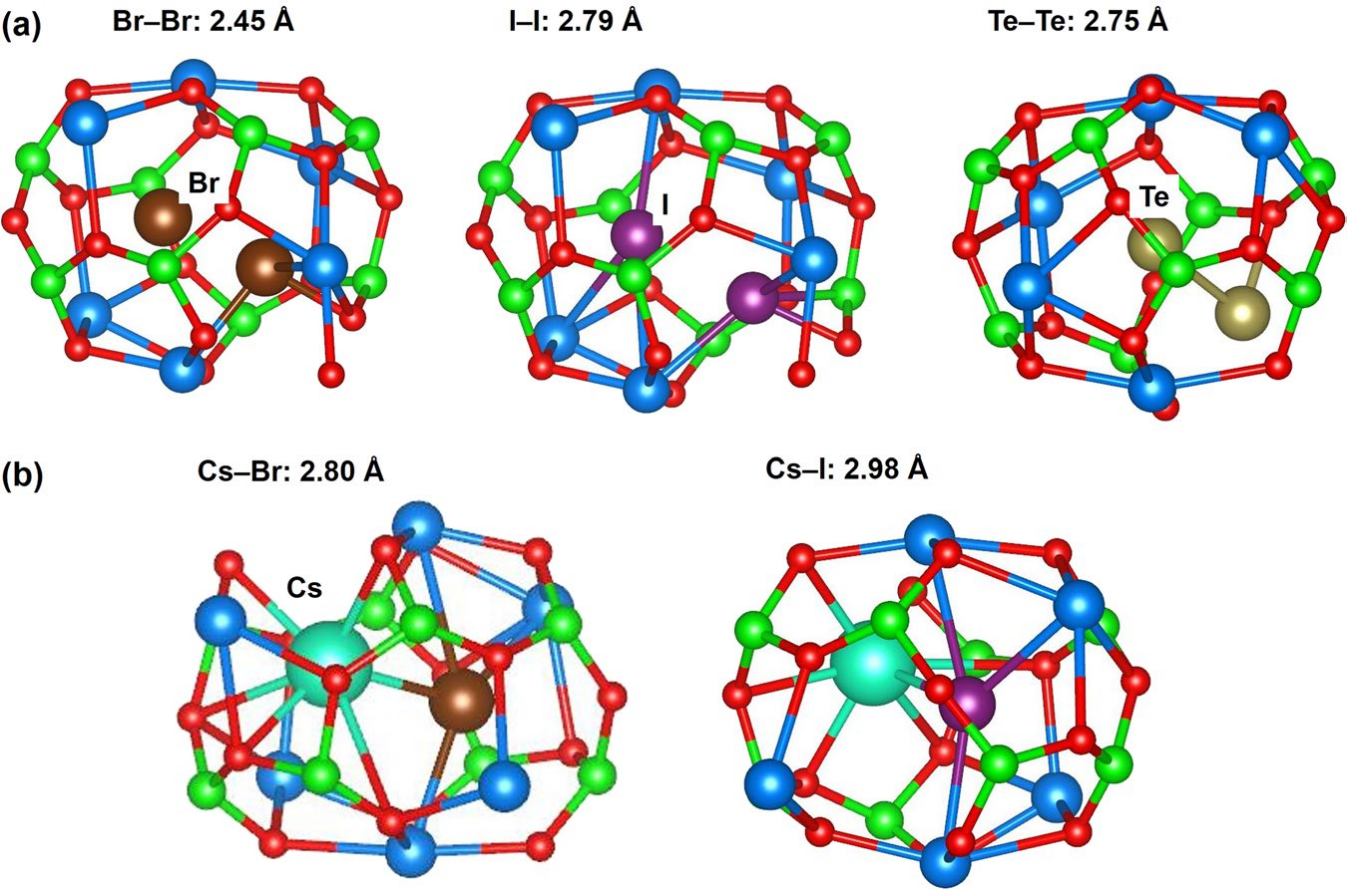
Relaxed structures of (**a**) Br_2_, I_2_ and Te_2_ and (**b**) CsBr and CsI encapsulated in a C12A7:O^2−^ cage.

The relaxed structures of dimers (homo and hetero) within a cage are shown in Fig. 6. The encapsulation energies and the Bader charges on the dimers are reported in Table 5. For all dimers, significant distortion is observed in cage walls and the molecules occupy positions with a halide close to the centre of the cage but the other species distinctly off-centre. The degree of distortion is reflected in the encapsulation energies. Furthermore, both homo and heteronuclear dimers are thermodynamically unstable.

**Table 5:** Encapsulation energies and Bader charges calculated for homonuclear and heteronuclear dimers encapsulated in a C12A7:O^2−^ cage.

Reaction	Encapsulation energy (eV/atom) with respect to dimer	Bader charge |e|	
		Br or I or Te	
Br_2_ + C12A7:O^2−^ → Br_2_·C12A7:O^2−^	1.48	−0.40, −0.06	
I_2_ + C12A7:O^2−^ → I_2_·C12A7:O^2−^	1.85	−0.41, +0.14	
Te_2_ + C12A7:O^2−^ → Te_2_·C12A7:O^2−^	1.10	+0.65, −0.58	
		Br or I	Cs
CsBr + C12A7:O^2−^ → CsBr·C12A7:O^2−^	2.26	−0.78	+0.69
CsI + C12A7:O^2−^ → CsI·C12A7:O^2−^	2.79	−0.69	+0.68

The bond distances of Br_2_ and I_2_ are 2.45 Å and 2.79 Å respectively, larger than their corresponding gas phase dimer values of 2.31 Å and 2.69 Å. This is partly because the overall Bader charges on Br_2_ and I_2_ are slightly negative meaning that if they were gas phase molecules there would be occupation of a more diffuse π^*^ molecular orbital. However, these molecules also show strong induced polarization, which in I_2_ means the atom near the centre of the cage has a negative charge whereas the other atom has a positive Bader charge.

In the case of Te_2_, there is an elongation of 0.16 Å in the bond length compared to the gas phase molecule. This elongation is similar to the values observed in Br_2_ and I_2_. However, the Te_2_ molecule is the most strongly polarized and has the smallest overall charge.

CsBr and CsI molecules also experience strong distortion and their encapsulation energies are even more unfavourable that those of the homonuclear dimers. The bond distances of CsBr and CsI in the cage are ~0.30 Å shorter than their corresponding calculated gas phase dimers of 3.09 Å and 3.34 Å respectively, presumably due to the confinement induced by the cage. Nevertheless, the Bader charge distribution is roughly symmetric with absolute values as expected in a gas phase molecule.

### Dimers occupying C12A7:e^−^

**Figure 7:**
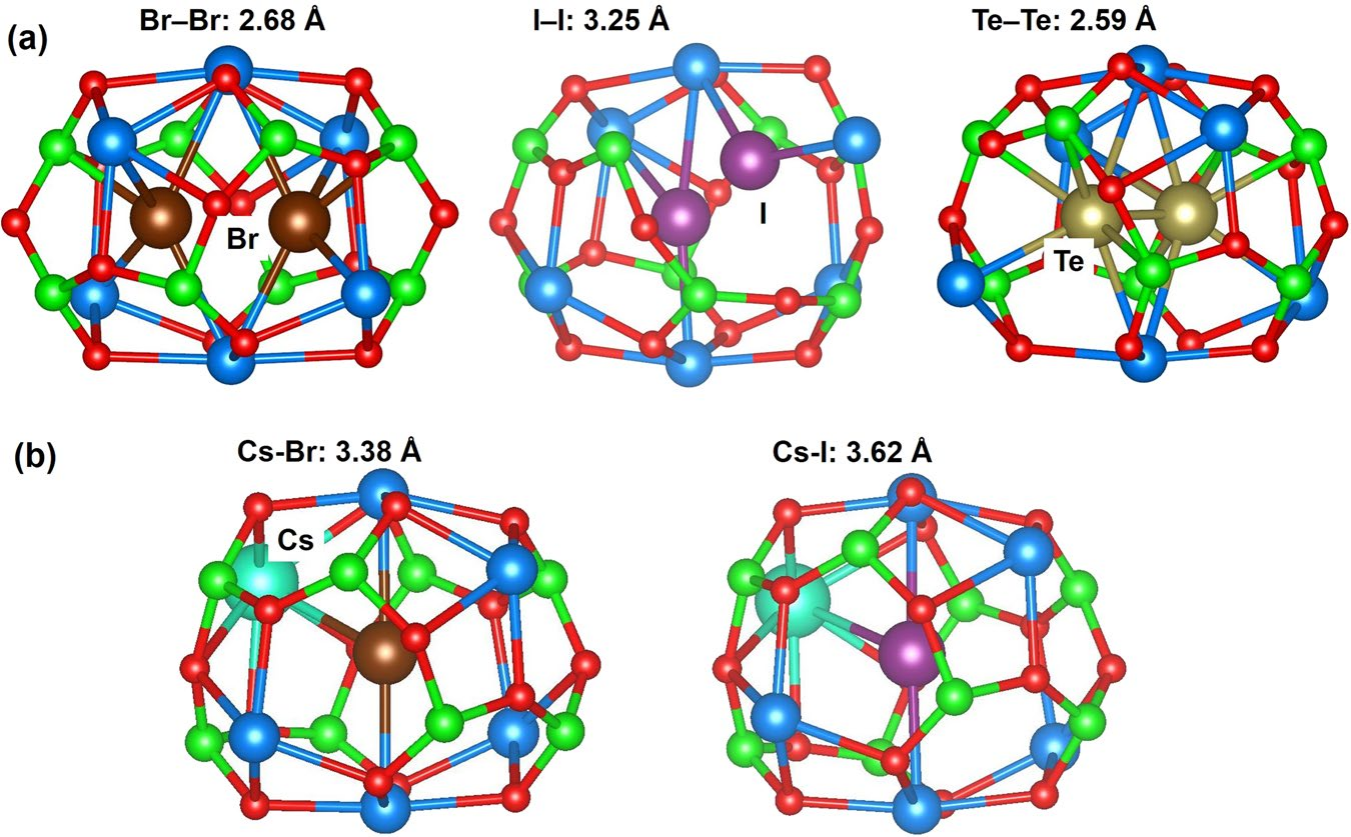
Relaxed structures of (**a**) Br_2_, I_2_ and Te_2_ and (**b**) CsBr and CsI encapsulated in C12A7:e^−^.

Finally we considered both homo and heteronuclear dimers inside C12A7:e^−^. The relaxed structures of dimers introduced inside C12A7:e^−^ are shown in Fig. 7. The encapsulation energies and the Bader charges on the dimers are reported in Table 6.

**Table 6:** Encapsulation energies and Bader charges calculated for homonuclear dimers encapsulated in C12A7:e^−^.

Reaction	Encapsulation energy (eV/atom) with respect to dimer	Bader charge |e|	
		Br or I or Te	
Br_2_ + C12A7:e^−^ → Br_2_·C12A7:e^−^	−1.00	−0.83 (2)	
I_2_ + C12A7:e^−^ → I_2_·C12A7:e^−^	+0.18	−0.69, −0.70	
Te_2_ + C12A7:e^−^ → Te_2_·C12A7:e^−^	+0.09	−0.67, −0.80	
		Br or I	Cs
CsBr + C12A7:e^−^ → CsBr·C12A7:e^−^	+1.52	−0.76	+0.69
CsI + C12A7:e^−^ → CsI·C12A7:e^−^	+1.60	−0.69	+0.73

The bond distance for Br_2_ inside a cage is 2.68 Å, longer than its corresponding gas phase dimer, 2.31 Å (but similar to calculations for C12A7:O^2−^). Nevertheless, the encapsulation energy for two Br atoms to occupy a cage with respect to an isolated dimer is exothermic (see Table 6) and thus the molecule is more stable in the cage. Quite differently to C12A7:O^2−^, the dimer sits symmetrically across the cage and the Bader charges (Table 6) show that both Br atoms gain electrons from the lattice to form Br^−^ ions (commensurate with a longer probably non-bonding Br-Br distance). While the encapsulation energy of Br_2_ is exothermic, with respect to an isolated Br_2_ molecule, the reaction to split the molecule into two cages is strongly exothermic (−2.91 eV). Thus, if there is an unoccupied cage available, the molecule will dissociate into two Br^−^ anions in separate cages. Furthermore, the energy to add ½ Br_2_ to a single existing Br^−^ ion in a single cage is 1.92 eV. Thus, even if no cage remains unoccupied by a Br^−^ anion, Br_2_ molecules within C12A7:e^−^ will still not form.

Conversely to Br_2_, there is significant distortion of a cage occupied by two I atoms and the molecule orientation is asymmetric (see Fig. 7a) although the Bader charges on the I anions are almost identical. The I_2_ bond distance is 3.25 Å, considerably longer than its corresponding gas phase dimer, 2.69 Å. This is reflected in the endothermic I_2_ encapsulation energy. Essentially the two I^−^ ions demand a larger volume than the cage can provide.

At first sight two Te atoms do seem to form a dimer in the cage, with the molecular distance 2.59 Å very close to the isolated gas phase bond distance of 2.58 Å. However, the Te atoms attract charge (over ½ an electron each), thus it is really a Te_2_^−^ or even a Te_2_^2−^ anion species. Despite attracting charge and the associated electron affinity gain, the encapsulation of Te_2_ is endothermic. This reflects the size of the molecule with respect to the cage volume.

Finally, we consider the encapsulation of a single heteronuclear diatomic molecule of CsBr or CsI inside a cage. The relaxed structures are shown in Fig. 7b. Calculated Cs-Br and Cs-I in-cage distances are 3.38 Å and 3.62 Å respectively. Both CsBr and CsI are ~0.3 Å longer than their corresponding calculated isolated values of 3.09 Å and 3.34 Å respectively. In the relaxed structures, Br or I occupy the centre of the cage but the Cs ion displaces one of the Ca^2+^ ions in the cage wall (see Fig. 7b), which then sits near the centre of an adjacent cage. The energies to encapsulate CsBr and CsI and the Bader charges are reported in Table 6; both are strongly endothermic. Essentially there is insufficient volume within the cage to accommodate those large molecules.

In conclusion, atomic scale simulations based on DFT have been employed to study the possibility of using C12A7 as a filter material in its stoichiometric and electride forms, encapsulating volatile fission products released during processing of spent nuclear fuel. Overall, C12A7 can selectively accommodate Br and I as single extraframework anions occupying upto one in three cages. Te is also strongly incorporated but only up to one in six cages.

More specifically, Xe, Kr, and Cs are unstable in both forms of C12A7. Rb shows a weak encapsulation in C12A7:O^2−^ but is not stable in C12A7:e^−^. Br, I and Te are stable as anions from atoms in both forms of C12A7, but, C12A7:e^−^ shows a significant enhancement in encapsulation energies due to the availability of extra-framework electrons. Successive encapsulation of Br, I and Te was considered so that multiple cages within a unit cell are occupied by these species. Successive encapsulation energies decrease with the addition of atoms in C12A7:O^2−^. However, in C12A7:e^−^, the encapsulation energies remain constant up to four consecutive encapsulation of Br (or I) or two Te atoms, due to the available electrons to be trapped by the additional atoms to form anions. Homonuclear (Br_2_, I_2_ and Te_2_) and heteronuclear dimers (CsBr and CsI) were allowed to occupy one of the cages in unit cells of C12A7:e^−^ and C12A7:O^2−^. Calculations show (except for Br_2_) that both homo and hetero nuclear dimers are thermodynamically unfavourable in C12A7:O^2−^ from their dimers since they introduce a significant distortion in the cage wall. While Br_2_ is stable as a molecular species, it is more stable as two anions occupying separate cages.

## Methods

The calculations were carried out using the spin-polarized mode of DFT as implemented in the VASP code^26,27^. The exchange-correlation term was modelled using the generalized gradient approximation (GGA) parameterized by Perdew, Burke, and Ernzerhof (PBE)^28^. In all cases we have used a plane-wave basis set with a cut off value of 500 eV and a 2 × 2 × 2 Monkhorst-Pack^29^ k-point mesh, which yields 8 k points. Further increase in the k points resulted in a total energy difference of 0.6 meV per atom. Structural optimizations were performed using a conjugate gradient algorithm^30^ and the forces on the atoms were obtained from the Hellman-Feynman theorem including Pulay corrections. In all optimized structures, forces on the atoms were smaller than 0.001 eV/Å and the stress tensor was less than 0.002 GPa. We define the encapsulation energy for single atoms or dimers trapped inside the empty cages of the electride form of C12A7 through the following equation;

1 $${{\rm{E}}}_{{\rm{Enc}}}={\rm{E}}({\rm{FP}}-{\rm{C}}12{\rm{A}}7:\,{{\rm{e}}}^{-})-{\rm{E}}({\rm{C}}12{\rm{A}}7:\,{{\rm{e}}}^{-})-{\rm{n}}\,{\rm{E}}({\rm{FP}})$$

where E (C12A7:e^−^) is the total energy for bulk C12A7:e^−^, E (FP-C12A7:e^−^) is the total energy of the gaseous atom or atoms occupying the cages, E(FP) is the total energy of an isolated fission product (the reference state) and n is the number of fission product atoms considered in the process.

The inclusion of van der Waals (vdW) interactions is particularly important for the incorporation of highly polarizable noble gas and halogen atoms. Here, dispersion has been included by using the pair-wise force field as implemented by Grimme *et al*.^31^ (DFT-D3) in VASP.

## Data Availability

The data that support the findings of this study are available from the corresponding author upon reasonable request.

## References

[1] Erfrmenkov VM (1989). IAEA Bulletin.

[2] Lee, W. E *et al*. In *Radioactive Waste Management and Contaminated Site Clean-Up* (Woodhead Publishing, 2013),p.xvii.

[3] Devisme, F., Juvenelle, A. & Touron, E. Strategy and current status of research on enhanced iodine separation during spent fuel reprocessing by the PUREX process, Sept. 9–13, Paris, France: Global’01 (2001).

[4] Yusan S, Erenturk S (2011). Adsorption Characterization of Strontium on PAN/Zeolite Composite Adsorbent. World Journal of Nuclear Science and Technology.

[5] Karhu, A. Methods to prevent the source term of methyl lodide during a core melt accident (NKS-13). Denmark (1999).

[6] Sava DF (2011). Capture of Volatile Iodine, a Gaseous Fission Product, by Zeolitic Imidazolate Framework-8. J. Am. Chem. Soc.

[7] Yao R-X, Cui X, Jia X-X, Zhang F-Q, Zhang X-M (2016). A Luminescent Zinc(II) Metal–Organic Framework (MOF) with Conjugated π-Electron Ligand for High Iodine Capture and Nitro-Explosive Detection. Inorg. Chem..

[8] Sava DF, Garino TJ, Nenoff TM (2012). Iodine Confinement into Metal–Organic Frameworks (MOFs): Low-Temperature Sintering Glasses To Form Novel Glass Composite Material (GCM) Alternative Waste Forms. Ind. Eng. Chem. Res.

[9] He J (2014). Immobilization of Volatile and Corrosive Iodine Monochloride (ICl) and I2 Reagents in a Stable Metal–Organic Framework. Inorg. Chem.

[10] Bartl H, Scheller T (1970). Zur Struktur des (CaO)_12_ (Al_2_O_3_)_7_. N. Jb. Miner. Mh..

[11] Imlach JA, Glasser LSD, Glasser FP (1971). Excess oxygen and the stability of 12CaO 7Al_2_O_3_. Cement Conc. Res..

[12] Watauchi S, Tanaka I, Hayashi K, Hirano M, Hosono H (2002). Crystal growth of Ca1_2_Al1_4_O_33_ by the floating zone method. J. Cryst. Growth.

[13] Taylor, H. F. W. Cement Chemistry, 2nd Edition, Thomas Telford, London (1997)

[14] Nurse RW, Welch JH, Majumdar AJ (1965). The 12CaO.7Al_2_O_3_ phase in the CaO.Al_2_O_3_ system. Trans. Br. Cerem. Soc..

[15] Hayashi K, Hirano M, Hosono H (2005). Thermodynamics and Kinetics of Hydroxide Ion Formation in 12CaO·7Al_2_O_3_. J. Phys. Chem. B.

[16] Matsuishi S (2003). High-Density Electron Anions in a Nanoporous Single Crystal: [Ca_24_Al_28_O_64_]^4+^(4e^−^). Science.

[17] Kim SW (2007). Metallic State in a Lime−Alumina Compound with Nanoporous Structure. Nano Lett..

[18] Jeevaratnam J, Glasser FP, Glasser LSD (1964). Anion Substitution and Structure of 12CaO·7Al_2_O_3_. J. Am. Chem. Soc.

[19] Zhmoidin GI, Chatterjee AK (1984). Conditions and mechanism of interconvertibility of compounds 12CaO.7Al_2_O_3_ and 5CaO.3Al_2_O_3_. Cem. Concr. Res.

[20] Hosono H, Abe Y (1987). Occurrence of superoxide radical ion in crystalline calcium aluminate 12CaO.7Al_2_O_3_ prepared via solid-state reactions. Inorg. Chem.

[21] Miyakawa M (2006). Photoluminescence from Au ion-implanted nanoporous single-crystal 12CaO•7Al_2_O_3_. Phys. Rev. B.

[22] Kitano M (2015). Electride support boosts nitrogen dissociation over ruthenium catalyst and shifts the bottleneck in ammonia synthesis. Nat. Commun.

[23] Kuganathan N, Hosono H, Shluger AL, Sushko PV (2014). Enhanced N_2_ Dissociation on Ru-Loaded Inorganic Electride. J. Am. Chem. Soc.

[24] Sushko PV, Shluger AL, Toda Y, Hirano M, Hosono H (2011). Models of stoichiometric and oxygen-deficient surfaces of subnanoporous 12CaO•7Al_2_O_3_. Philos. Trans. Royal Soc. A.

[25] Lide, D. R. CRC Handbook of Chemistry and Physics. 86th ed. Boca Raton, FL: CRC (2005).

[26] Kresse G, Furthmüller J (1996). Efficient iterative schemes for ab initio total-energy calculations using a plane-wave basis set. Phys. Rev. B.

[27] Kresse G, Joubert D (1999). From ultrasoft pseudopotentials to the projector augmented-wave method. Phys. Rev. B.

[28] Perdew JP, Burke K, Ernzerhof M (1996). Generalized Gradient Approximation Made Simple. Phys. Rev. Lett.

[29] Monkhorst HJ, Pack JD (1976). Special points for Brillouin-zone integrations. Phys. Rev. B.

[30] Press, W. H., Flannery, B. P., Teukolsky, S. A. & Vetterling, W. T. Numerical Recipes. The Art of Scientific Computing, Cambridge Univ. Press, Cambridge, 818 pp (1986).

[31] Grimme S, Antony J, Ehrlich S, Krieg H (2010). A consistent and accurate ab initio parametrization of density functional dispersion correction (DFT-D) for the 94 elements H-Pu. J. Chem. Phys..

[32] D.R. Lide CRC Handbook of Chemistry and Physics (86th ed.), CRC, Boca Raton, FL (2005).

